# Changing minds and methods: providing health sciences faculty with alternatives to systematic reviews assignments

**DOI:** 10.5195/jmla.2026.2056

**Published:** 2026-01-01

**Authors:** Laura Lipke, Neyda Gilman

**Affiliations:** 1 llipke@binghamton.edu, Binghamton University, Binghamton, NY; 2 ngilman@binghamton.edu, Binghamton University, Binghamton, NY

**Keywords:** Health Sciences, Evidence Synthesis, Systematic Review, Research Instruction, Graduate assignments, Cognitive load theory

## Abstract

**Background:**

Health sciences librarians frequently engage in discussions about the appropriate assignment of evidence synthesis reviews (ES) for graduate students as course, thesis, or capstone projects. Such reviews are often assigned to build the research skills needed in a clinical environment. In the assignment of these reviews, it has become apparent that health sciences faculty are often not familiar with required standardized methodologies. Incorrect methodologies can contribute to research waste and produce evidence that cannot be applied for its intended purpose.

**Case Presentation:**

Health sciences librarians at an R1 institution ventured to address the ES review knowledge gap through a continuing education webinar for health sciences faculty and graduate students. The webinar provided guidance on systematic review (SR) methodology, optional alternative research assignments, and discussions encouraging the use of these assignments. The alternative assignments were developed based on those presented by Lipke & Price (2025), each with specific learning objectives and grading rubrics. Pre- and post-webinar surveys were conducted to gauge any changes in participants' knowledge, skills, or abilities related to the presented information.

**Conclusions:**

Study participants included six faculty and a graduate student. Survey results showed that participants had an improved understanding of, and placed increased importance on, ES method guidelines, with an equal understanding of the need for alternative assignments. The authors of this study will further evaluate the impact of this webinar and assess its effectiveness in changing health sciences research assignments.

## BACKGROUND

Concerns regarding the quality and sheer number of published evidence synthesis (ES) reviews, especially systematic reviews (SR), in the health sciences is well documented in recent scholarship [1–10]. There are also a number of publications supporting and refuting the inclusion of ES reviews as graduate and doctoral program capstone or thesis projects [11–16]. Those that refute the inclusion of reviews highlight the lack of knowledge, skills, and mentoring in the rigorous methodology required to conduct the reviews and suggest that faculty need to update their knowledge of these methodologies prior to incorporating such assignments into the curriculum [11–19]. Although the skills learned from conducting ES reviews are essential to students, alternative learning methods are clearly needed due to inconsistent levels of mentorship available [12,14–16,19–22]. The field of health sciences librarianship is well aware of the obstacles faced by these students when assigned such reviews and the frustration involved with balancing deliverable requirements set by faculty and the expectations established by standardized conduct guidelines [23].

In response to their concerns and the growing popularity of ES in general, health sciences librarians often provide ES review methodology consultations and instruction sessions. While many versions of these are provided in the scholarship, the majority are developed for students with fewer developed for practitioners or instructors [24–27]. A review of the literature identified that even fewer, if any of the ES information sessions, were specifically focused upon educating the faculty that assign these reviews to their students. Of closest note was a three day seminar provided by an academic library to improve reproducibility and a librarian-led webinar on data literacy for a faculty learning community [28,29].

Building off of this research, in an attempt to remedy the issues the methodology knowledge gap and the assigning of ES reviews in health sciences curriculum, the authors of the article Rethinking Systematic Review Assignment Design in Graduate Health Sciences Education from Librarians’ Perspectives presented modified ES assignments, based on the Cognitive Load Theory (CLT) of Chunking, to guide faculty and students through the process in a manageable fashion [30]. The authors suggested that future research surrounding these modified ES assignments be conducted through information sessions such as webinars where health sciences faculty are introduced to the assignments. It was recommended that this webinar begin by providing the faculty with an overview of the standardized methodology for ES reviews in order to facilitate their understanding of the complexities involved. The modified assignments could then be introduced as a way to provide students with research experiences that are achievable and promote learning [30].

Providing such webinars would be an opportunity for librarians to broaden their faculty outreach initiatives. While general librarian-faculty outreach is a common practice in academic librarianship that benefits both the faculty members and the librarians, few, if any, outreach initiatives document instructional sessions specifically designed for faculty [29,31]. Despite this, recent research has found that many Nursing faculty researchers are interested in attending research related webinars [31].

This case study evaluated the effectiveness of combining outreach and education through a webinar designed for health sciences faculty. The webinar provided attendees with the knowledge of standardized ES conduct guidelines and methods, helping them to provide students with achievable research assignments in lieu of the full systematic reviews regularly assigned in graduate programs. Pre and post webinar surveys were used to measure the change of faculty participants’ knowledge, skills, and attitudes toward the adoption of alternative modified systematic review assignments in the health sciences graduate program curriculum.

## CASE PRESENTATION

Binghamton University is an R1 state institution with health sciences programs of nursing, physical therapy, occupational therapy, speech and language pathology, health and wellness, public health, and pharmacy. Two librarians share liaison responsibilities for these programs and both have noted that students and faculty frequently confuse the methodologies of various types of reviews and are unaware of the standardized methodology guidelines (eg. Cochrane or JBI) required for SRs. To address these issues, they were inspired by an experienced SR librarian and author to use the method of chunking to develop alternative assignments that could be completed by a single student or group of students, within the time span of a semester [32]. These chunked assignments provide students and faculty with projects that would challenge the students to learn the necessary SR methodological guidelines in a way that encourages a successful experience. The information provided with these assignments includes learning objectives and standardized forms to use as rubrics.

### Alternative Assignments

The alternative assignments to be presented in this webinar are based on those designed by Lipke and Price, the Cochrane and JBI guidelines, and the reporting standards of PRISMA [30,33–35]. Each assignment may be applied to build a completed review or used as independent assignments. First is a narrative review which through its objectives encourages the understanding of the purpose of this type of review and how it provides topic background and identifies scholarship surrounding the topic with the intent of identifying a research gap.

The peer review assignment may be used with the narrative review or protocol assignments. The peer review assignment prepares students for scholarly publishing and how to incorporate critique into their final manuscript. The objectives for this assignment guide the student to learn about the peer review process, grow from constructive feedback and learn the required elements of PRISMA-P [36].

The protocol assignment, associated objectives, and grading rubric follow the PRISMA-P reporting standards [36]. The purpose of a SR protocol is to establish a detailed plan for the review project and to reduce bias during the screening and data extraction phases. This assignment introduces the learner to the steps of a SR, the requirements of PRISMA-P and encourages them to thoughtfully plan the details of the review.

The search methods exercise and its objectives are based on the PRISMA-S [37]. The purpose of this assignment is to introduce the development of search strategies combining keywords and controlled vocabulary, the required reporting standards, and how these standards improve the transparency and reproducibility of the review. The PRISMA-S may be used as a grading rubric.

The critical appraisal assignment emphasizes the importance of unbiased and reproducible SR methodology and introduces the critical appraisal stage of SRs. The JBI or Critical Appraisal Skills Programme (CASP) critical appraisal checklists are used to guide the learner to critique a review and meet objectives such as the importance of critical appraisal, methodological rigor and critical thinking skills. These checklists may also be used as grading rubrics [38,39].

The data extraction exercise is based on chapter 5 of the Cochrane handbook and can be completed with qualitative and/or quantitative data [33]. Student learning objectives are developed on the requirements of using the pre-established inclusion/exclusion criteria to guide the extraction phase of a review as well as the importance of transparent and reproducible methods.

The last three alternative assignments, a systematized review, updating an existing review, and a rapid review require the learner to complete all steps of a review but in modified fashions. A systematized review includes all of the elements of SRs, but does not meet all requirements for rigorous evidence evaluation or publication [40]. Updating a SR requires an initial critical appraisal of the review to ensure a rigorous methodology baseline then continues from the last date of the previous search, with fewer results and less screening and data extraction than a full review. The rapid review modifies some stages to shorten the timeline. These modifications are documented in detail to ensure transparency. The objectives of these assignments follow the guidelines for conducting such reviews as documented in the Cochrane handbook [33].

Further details of each assignment have been published elsewhere [30] and can be found in osf.io: https://tinyurl.com/ChangingMindsMethods.

### Webinar

The health sciences librarians designed a webinar for faculty and graduate students to promote the alternative SR assignments and provided continuing education credits for Nursing faculty. The primary goal of the two-hour webinar was to encourage the use of alternative assignments by enhancing attendees' knowledge, skills, and attitudes toward ES methodologies and educate faculty researchers.

With IRB approval, the librarians developed pre and post surveys to measure the webinar's effectiveness on participant's perceptions of the SR process and adoption of the alternative assignments. The pre survey was sent to registrants via a Qualtrics email and the post survey was provided at the end of the webinar via a link and QR code. The surveys featured a series of two demographic questions, eleven Likert-type questions related to pre and post knowledge, skills, and attitudes toward the standardized methodologies of SRs, participant opinions about the use of SRs as assignments, and one open-ended question [40].

The webinar was designed to have three phases: an introduction to ES definitions and methodologies, small group discussions about the pre-webinar learning activity and an introduction to the alternative assignments. Prior to the webinar, participants were provided with a SR and a critical appraisal worksheet as a pre-activity. The purpose of the activity was to introduce the required elements of SRs through critical appraisal and demonstrate the learning benefits of a chunked activity. Small group discussions allowed participants to discuss their appraisals and how they might do things differently. Lastly, participants were introduced to the chunked alternative assignments. All materials related to the webinar can be found in the osf.io repository files: https://tinyurl.com/ChangingMindsMethods.

### Workshop Evaluation Results

There were 7 participants in the webinar, 6 full time faculty (4 nursing, 1 physical therapy, and 1 occupational therapy) and 1 graduate nursing student. Data was tallied from 7 pre-surveys and 7 post-surveys (Table 1). The post survey was only shared in the webinar; it is therefore probable that the webinar participants are the same 7 who completed the post survey. However, due to the anonymity of the surveys, the authors are unable to match the pre- and post-surveys.

**Table 1:** Pre- and Post Survey Results, n=7

Survey Question	Pre-Survey			Post-Survey		
Have you authored a published systematic review?	No:	5	(71.4%)	No:	6	(85.7%)
	Yes:	2	(28.6%)	Yes:	1	(14.3%)
How would you rate your knowledge of systematic review methodology?	No Knowledge:	0	(0.0%)	No Knowledge:	0	(0.0%)
	Some Knowledge:	6	(85.7%)	Some Knowledge:	5	(71.4%)
	Expert Knowledge:	1	(14.3%)	Expert Knowledge:	2	(28.6%)
How would you rate your skills in performing a systematic review?	No Skills:	0	(0.0%)	No Skills:	0	(0.0%)
	Some Skills:	6	(85.7%)	Some Skills:	5	(71.4%)
	Expert Skills:	1	(14.3%)	Expert Skills:	2	(28.6%)
How important do you think it is to use the standardized guidelines to conduct and report a systematic review?	Not Important:	0	(0.0%)	Not Important:	0	(0.0%)
	Moderately Important:	0	(0.0%)	Moderately Important:	0	(0.0%)
	Extremely Important:	7	(100.0%)	Extremely Important:	7	(100.0%)
How would you rate your knowledge of matching the type of research question to the type of literature review?	No Knowledge:	0	(0.0%)	No Knowledge:	1	(14.3%)
	Some Knowledge:	6	(85.7%)	Some Knowledge:	4	(57.1%)
	Expert Knowledge:	1	(14.3%)	Expert Knowledge:	2	(28.6%)
How would you rate your skill level of matching the type of research question to the type of literature review?	No Skills:	0	(0.0%)	No Skills:	1	(14.3%)
	Some Skills:	6	(85.7%)	Some Skills:	5	(71.4%)
	Expert Skills:	1	(14.3%)	Expert Skills:	1	(14.3%)
How important do you think it is to match a specific type of research question to the research methodology?	Not Important:	0	(0.0%)	Not Important:	0	(0.0%)
	Moderately Important:	4	(57.1%)	Moderately Important:	2	(28.6%)
	Extremely Important:	3	(42.9%)	Extremely Important:	5	(71.4%)
How would you rate your knowledge of how to critically appraise a systematic review?	No Knowledge:	0	(0.0%)	No Knowledge:	1	(14.3%)
	Some Knowledge:	5	(71.4%)	Some Knowledge:	4	(57.1%)
	Expert Knowledge:	2	(28.6%)	Expert Knowledge:	2	(28.6%)
How do you rate your skills of how to critically appraise a systematic review?	No Skills:	1	(14.3%)	No Skills:	0	(0.0%)
	Some Skills:	5	(71.4%)	Some Skills:	6	(85.7%)
	Expert Skills:	1	(14.3%)	Expert Skills:	1	(14.3%)
How important do you think it is to critically appraise a systematic review before applying the conclusions of that review?	Not Important:	0	(0.0%)	Not Important:	0	(0.0%)
	Moderately Important:	2	(28.6%)	Moderately Important:	1	(14.3%)
	Extremely Important:	5	(71.4%)	Extremely Important:	6	(85.7%)
Do you think that a systematic review is appropriate for a single student, 12-week assignment?	Definitely Not:	1	(14.3%)	Definitely Not:	5	(71.4%)
	Might or Might Not:	6	(85.7%)	Might or Might Not:	1	(14.3%)
	Definitely Yes:	0	(0.0%)	Definitely Yes:	1	(14.3%)

All participants indicated a desire to learn more in the pre-survey open ended question. Reasons included wanting to gain “more knowledge of systematic reviews and their place in the curriculum,” getting “a better understanding of how to perform a systematic review,” or simply “more options.” While participants consistently rated the importance of following SR guidelines as extremely important, opinions on matching research questions to review types and the appropriateness of SRs for a 12-week student assignment shifted post-webinar, with more participants emphasizing the need to match questions and reviews and questioning SR's suitability for such assignments. Discussions during the webinar suggest confusion and misunderstandings about SRs, and ES more generally, that became better understood by the end of webinar. One example of this included confusion as to why dates of searches need to be documented with participants noting that the searching would be done, and updated, over time. After discussions, everyone understood the importance of documenting the date of the final searches. Similar discussions occurred around the various types of reviews, use of grey literature, protocol registration, PRISMA, and inclusion and exclusion criteria.

To further evaluate participants' thoughts, they were asked how they would apply what they had learned. Responses included applying this new knowledge to future research and course development and others wanted to expand their learning of the guidelines and grey literature.

## DISCUSSION

The assignment of systematic reviews within health sciences graduate programs, especially as a course deliverable, is a clear indication of the faculty knowledge gap regarding the complexity of ES review methodology. Although literature equally supports and refutes the inclusion of SRs for graduate capstone or thesis projects, anecdotal evidence experienced daily by health sciences librarians supports the need for further education for those mentoring graduate students through the review process [11–16]. Many librarian-led ES methodology instruction sessions are specifically designed for students [24–27]. Although Nursing faculty have expressed interest in research webinars when surveyed and a large percentage stated that they interact with the library for their research needs [31], we know of no publications describing ES methodology webinars for health sciences faculty. In an effort to further engage with the health sciences around ES, librarians at this institution reached out to faculty proposing an ES methodology continuing education webinar, which they accepted.

The webinar was designed to enhance faculty understanding of rigorous ES methodologies and promote alternative SR assignments. Positive movement was made on both of these goals. One note of interest is that the pre-and post-surveys showed a decrease from two to one in the number of people indicating they had published a SR. This may be due to different individuals filling out the polls or could demonstrate improved understanding of SRs. The discussions and results of the study highlighted the willingness of health sciences faculty to consider alternative ES assignments when educated in required methodologies. The number of participants who thought SRs were appropriate for a single student, 12-week assignment decreased after the webinar. The open-ended survey responses included desires to learn more about SRs and to apply the lessons from the webinar to future curriculum and research. Overall, participants showed an increased understanding, positive shift in perceptions, and readiness to implement the assignment alternatives suggesting a promising approach to improving ES educational practices within graduate programs.

## LIMITATIONS AND FUTURE PLANS

The primary limitations of the study are its small sample size from one institution, the majority of nursing participants, and its inability to measure specific participant responses from anonymous surveys. Future webinars will link pre- and post-surveys, tying responses together. Despite these limitations, the study provides a stepping stone for health sciences librarians to suggest and promote the use of alternative SR assignments.

Based on the discussions within and around the webinar, and the open-ended survey responses, the authors are encouraged to continue this work. The authors will reach out to the participants after a full academic year through an anonymous survey designed to assess participants’ claimed plans to apply what they have learned. This would be beneficial to see if the lessons from the webinar have been maintained, and to further promote the alternative assignments. Continued engagement with participants could strengthen the already solid relationship between the programs and the library and help the authors improve future webinars and communications around ES.

The webinar occurred during a spring semester and the authors intend to offer the same webinar again in a fall semester then offered annually and adjusted to fit the growing needs of the health sciences programs. The collaboration with nursing in providing accredited continuing education credits will continue as feasible. These credits were likely an additional motivation for participants to attend. The authors will work to expand the reach of this webinar to other departments and explore additional promotional avenues. Faculty/liaison interactions will continue to address SR related assignment and methodology questions. Increased promotion of the recently created ES LibGuide (https://libraryguides.binghamton.edu/literaturereview) is also planned. Future research will explore sustained implementation and broader impacts across diverse educational settings to further validate this study's findings and inform best practices in health sciences education.

## CONCLUSION

The challenges created by the rapid growth of ES products, including the quality of published SRs, have been a growing concern of health sciences librarians. Librarians frequently support individuals who may not be familiar with the complexities of ES or the importance of adhering to proper methodologies. Academic librarians can work towards improving the ES knowledge and skills of the faculty and students at their institutions through webinars, library guides, and alternative assignments similar to those discussed in this article.

The growing demand for SRs as capstone projects in health sciences graduate programs underscores the need to equip both students and faculty with alternative methods to learn how to conduct rigorous, evidence-based research. This study demonstrates that faculty gained a better understanding of SR methodology through a targeted webinar, revealing a positive shift in attitudes and a desire to incorporate proposed alternative assignments into future curricula. The findings suggest that health sciences faculty are open to collaborating with librarians to redesign SR assignments, provided they receive guidance on best practices and methodological rigor.

Looking ahead, ongoing faculty engagement, webinar expansion, and research on long-term impacts will refine ES education to better prepare students for evidence-based healthcare research. Future work will focus on further developing the alternative assignments, assessing their impact, and promoting their adoption across health sciences programs. This study provides a foundation for future librarian-driven efforts to enhance the quality and effectiveness of ES education in graduate curricula through the application of cognitive load theory and engagement with faculty.

## Data Availability

All data associated with this study are available in the Open Science Framework at: https://osf.io/pnjsf/.

## SUPPLEMENTAL FILES

**Appendix A:** A recording of the webinar can be found in the Binghamton University institutional repository: (https://orb.binghamton.edu/library_resources/31/).
